# Alterations in mitochondrial dynamics with age‐related Sirtuin1/Sirtuin3 deficiency impair cardiomyocyte contractility

**DOI:** 10.1111/acel.13419

**Published:** 2021-07-03

**Authors:** Jingwen Zhang, Zhibin He, Julia Fedorova, Cole Logan, Lauryn Bates, Kayla Davitt, Van Le, Jiayuan Murphy, Melissa Li, Mingyi Wang, Edward G. Lakatta, Di Ren, Ji Li

**Affiliations:** ^1^ College of Life Sciences Shandong Normal University Jinan China; ^2^ Department of Surgery Morsani College of Medicine University of South Florida Tampa FL USA; ^3^ Laboratory of Cardiovascular Science Intramural Research Program National Institute on Aging National Institutes of Health Baltimore MD USA

**Keywords:** aging, ischemia/reperfusion, mitochondria fission and fusion, SIRT1, SIRT3

## Abstract

Sirtuin1 (SIRT1) and Sirtuin3 (SIRT3) protects cardiac function against ischemia/reperfusion (I/R) injury. Mitochondria are critical in response to myocardial I/R injury as disturbance of mitochondrial dynamics contributes to cardiac dysfunction. It is hypothesized that SIRT1 and SIRT3 are critical components to maintaining mitochondria homeostasis especially mitochondrial dynamics to exert cardioprotective actions under I/R stress. The results demonstrated that deficiency of SIRT1 and SIRT3 in aged (24–26 months) mice hearts led to the exacerbated cardiac dysfunction in terms of cardiac systolic dysfunction, cardiomyocytes contractile defection, and abnormal cardiomyocyte calcium flux during I/R stress. Moreover, the deletion of SIRT1 or SIRT3 in young (4–6 months) mice hearts impair cardiomyocyte contractility and shows aging‐like cardiac dysfunction upon I/R stress, indicating the crucial role of SIRT1 and SIRT3 in protecting myocardial contractility from I/R injury. The biochemical and seahorse analysis showed that the deficiency of SIRT1/SIRT3 leads to the inactivation of AMPK and alterations in mitochondrial oxidative phosphorylation (OXPHOS) that causes impaired mitochondrial respiration in response to I/R stress. Furthermore, the remodeling of the mitochondria network goes together with hypoxic stress, and mitochondria undergo the processes of fusion with the increasing elongated branches during hypoxia. The transmission electron microscope data showed that cardiac SIRT1/SIRT3 deficiency in aging alters mitochondrial morphology characterized by the impairment of mitochondria fusion under I/R stress. Thus, the age‐related deficiency of SIRT1/SIRT3 in the heart affects mitochondrial dynamics and respiration function that resulting in the impaired contractile function of cardiomyocytes in response to I/R.

## Graphical abstract

Cardiac SIRT1 and SIRT3 are decreased with age. The age‐related SIRT1/SIRT3 deficiency causes impaired contractile function of cardiomyocytes due to mitochondrial dysfunction.

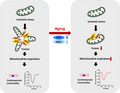

**Abbreviations**
AMPK: Adenosine monophosphate‐activated protein kinase
ATP: Adenosine triphosphate
ATP5A: Mitochondrial F1 Complex Alpha Polypeptide
ECG: Electrocardiogram
FOXO3: Forkhead box O 3
H/R: Hypoxia/reoxygenation
I/R: Ischemia/reperfusion
LAD: Left anterior descending
LKB1: Liver kinase B1
MCU: Mitochondrial calcium uniporter
MFN1: Mitofusion 1
MFN2: Mitofusion 2
MTCO1: Mitochondrially Encoded Cytochrome C Oxidase I
mtDNA: Mitochondria DNA
NAD^+^
: Nicotinamide adenine dinucleotide
NDUFB8: NADH dehydrogenase [ubiquinone] 1 beta subcomplex subunit 8
OCR: Oxygen consumption rate
OPA1: Optic atrophy 1
OXPHOS: Oxidative phosphorylation
ROS: Reactive oxygen species
SDHB: Succinate dehydrogenase subunit B
Sir2: Silent information regulator 2
SIRT1: Sirtuin 1
SIRT3: Sirtuin 3
TEM: Transmission electron microscopy
UQCRC2: ubiquinol cytochrome c reductase core protein II

## 1 INTRODUCTION

The mammalian sirtuins are a family of highly conserved nicotinamide adenine dinucleotide (NAD)^+^‐dependent deacetylases with homology to silent information regulator 2 (Sir2) (Imai & Guarente, 2010). Increasing studies prompt the key role of sirtuins in the extension of lifespan (Zhao et al., 2020). Sirtuin1 (SIRT1) is the closest mammalian homolog to the yeast Sir2 protein in sequence and is expressed widely in tissues such as the liver, heart, and muscle of mice (Imai & Guarente, 2010; Lavu et al., 2008). Sirtuin3 (SIRT3) is expressed at a high level in the tissues with high metabolic turnover and mitochondrial content (Sun et al., 2018). Both SIRT1 and SIRT3 act pivotal parts in modulating several processes ranging from cell survival to energy homeostasis that show putative beneficial effects associated with their activation concerning their role in the lifespan extension (Gu et al., 2013; Porter et al., 2014).

In the heart, ischemia/reperfusion (I/R) stress markedly decreases the contractile activity (Duncker et al., 1998), especially in the aging hearts (Fares & Howlett, 2010). The myocardium is in high demand for energy to maintain the cardiac contractions (Muir & Hamlin, 2020). Mitochondria are the main energy producer in cardiomyocytes for contractile function by mitochondria oxidative phosphorylation (OXPHOS) (Bers, 2002). As highly dynamic organelles, mitochondria process adaptive alterations in shape and ultrastructure in responding to cellular stress (Pernas & Scorrano, 2016). Thus, it is critical to keep mitochondria in a relatively stable functioning state via quality control mechanisms (i.e., fission and fusion) in response to I/R stress especially in cardiac aging (Maneechote et al., 2017).

SIRT1 and SIRT3 play a critical role in cardiac physiology and pathology and their deficiencies in senescence hearts increase the susceptibility of hearts to I/R (Parodi‐Rullan et al., 2017; Porter et al., 2014; Wang et al., 2018). These findings indicate that the role of SIRT1 and SIRT3 is indispensable in aged hearts in response to I/R stress. Moreover, SIRT1 deacetylates mitofusin 1 (MFN1) and mitofusion 2 (MFN2), which controls its stability and contributes to the mitochondria elongation under hypoxic condition (Oanh et al., 2017; Sooyeon et al., 2016). SIRT3 can directly deacetylate optic atrophy 1 (OPA1) to increase mitochondrial fusion levels, and deacetylation of forkhead box O 3 (FOXO3) by SIRT3 can promote MFN2 expression, thereby repairing the imbalance of mitochondrial fission and fusion and maintaining mitochondrial function (Samant et al., 2014; Tseng et al., 2013). However, it remains unknown whether the role of SIRT1 and SIRT3 is of significance to control mitochondria quality control in the context of myocardial I/R stress.

In this study, we determined that SIRT1 and SIRT3 are impaired with cardiac aging and their deficiency leads to contractile dysfunction in the myocardium during I/R stress. In addition, SIRT1 and SIRT3 are crucial to maintain mitochondrial OXPHOS integrity, modulate target proteins deacetylation, and preserve mitochondria DNA (mtDNA) content to protect the cardiac mitochondria respiration function under I/R stress. We demonstrate that deficiency of age‐related SIRT1 and SIRT3 induces impairment in cardiac mitochondria dynamics, leading to exacerbated myocardial reactive oxygen species (ROS) level upon I/R stress. Our results provide novel mechanistic insights into the roles of SIRT1 and SIRT3 in protecting cardiomyocytes against I/R injury.

## 2 RESULTS

### 2.1 Age‐related decline of SIRT1 and SIRT3 causes the impairment of AMPK activation during myocardial I/R

**FIGURE 1:**
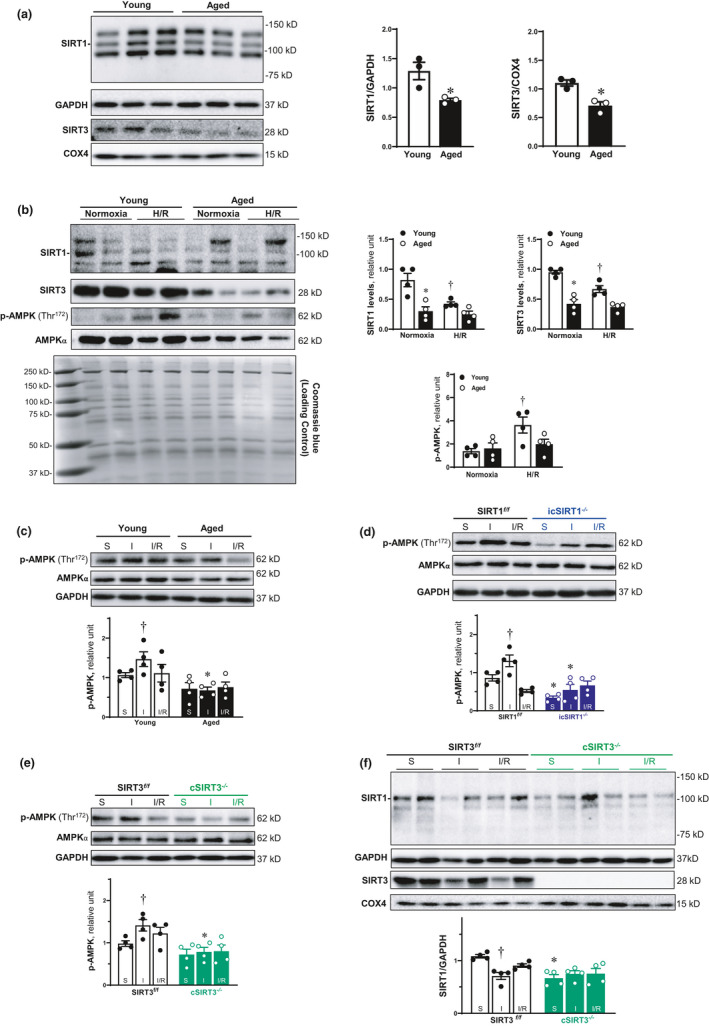
Age‐related decline of SIRT1 and SIRT3 causes the impairment of AMPK activation during myocardial I/R. (a) The protein expression level of SIRT1 and SIRT3 was decline in the left ventricle of aged (24–26 months) C57BL/6J hearts. Values are mean ± SEM. *N* = 3, *p < 0.05 vs. young (4–6 months) C57BL/6J hearts. (b) Expression of SIRT1 and SIRT3 in protein level was reduced with aging in the isolated cardiomyocytes. Their protein levels were decline in response to H/R in the cardiomyocytes of young hearts, and this stress‐related response was blunted in aged hearts. Values are mean ± SEM. *N* = 4, *p < 0.05 vs. young; ^†^ *p* < 0.05 vs. normoxia. (c) 30‐min ischemia and 6‐h reperfusion could activate AMPK, and the AMPK phosphorylation was impaired in aged hearts during I/R. Values are mean ± SEM. *N* = 4, *p < 0.05 vs. young; ^†^ *p* < 0.05 vs. sham. (d) The AMPK phosphorylation induced by cardiac I/R was blunted in the left ventricle of icSIRT1^−/−^ (4–6 months) hearts. Values are mean ± SEM. *N* = 4, *p < 0.05 vs. SIRT1*^f/f^* (4–6 months); ^†^ *p*<0.05 vs. sham. (e) The activation of AMPK during myocardial I/R was impaired in the left ventricle of cSIRT3^−/−^ (4–6 months) hearts. Values are mean ± SEM. *N* = 4, *p<0.05 vs. SIRT3*^f/f^* (4–6 months); ^†^ *p*<0.05 vs. sham. (f) The protein level of SIRT1 was decreased during myocardial ischemia. Cardiomyocyte‐specific deletion of SIRT3 caused the reduction of SIRT1 expression in the left ventricle of cSIRT3^−/−^ hearts. Values are mean ± SEM. *N* = 4, *p<0.05 vs. SIRT3*^f/f^*; ^†^ *p*<0.05 vs. sham.

To investigate the critical role of SIRT1 and SIRT3 in aged‐related ischemic heart disease, we first evaluated the protein expression level of SIRT1 and SIRT3 in the young (4–6 months) and aged (24–26 months) male C57BL/6J mice left ventricle. The immunoblotting results showed that both SIRT1 and SIRT3 expression levels were decreased with cardiac aging (Figure 1a).

Both SIRT1 (Wang et al., 2018) and SIRT3 (Huh et al., 2016) play an important role in regulating the activation of adenosine monophosphate (AMP)‐activated protein kinase (AMPK), which is a critical factor to protect cardiomyocyte mitochondrial function from I/R injury (Zaha et al., 2016). We detected the phosphorylation of AMPK and protein level of SIRT1 and SIRT3 in isolated cardiomyocytes from young (4–6 months) and aged (24–26 months) hearts in response to hypoxia/reoxygenation (H/R) stress. The SIRT1 and SIRT3 protein levels were downregulated with aging in the isolated cardiomyocytes (Figure 1b). Moreover, the H/R stress‐triggered AMPK phosphorylation in young cardiomyocytes but not in aged cardiomyocytes, suggesting that the age‐related deficiency of SIRT1 and SIRT3 contributes to the impairment of AMPK activation during H/R stress.

Furthermore, we performed the left anterior descending (LAD) coronary artery ligation in mouse model to examine the direct involvement of SIRT1 and SIRT3 during myocardial I/R. The results demonstrated that 30 min of ischemia and 6 h of reperfusion activated AMPK in young hearts, which was blunted in aged hearts (Figure 1c). The deficiency of SIRT1 and SIRT3 resulted in a marked reduction of phosphorylation levels of AMPK during ischemia (Figure 1d,e). These data suggest that age‐related reduction of SIRT1 and SIRT3 could cause an impaired regulation of AMPK phosphorylation at Thr^172^ of α catalytic subunit in response to cardiac I/R stress.

We have found that the hyperacetylation of AMPK upstream liver kinase B1 (LKB1) in aged and icSIRT1^−/−^ hearts during I/R contributes to the impaired ischemic AMPK activation (Wang et al., 2018). To understand the reason for an impaired ischemic AMPK activation occurring in cSIRT3^−/−^ hearts, we assessed the SIRT1 protein level in cSIRT3^−/−^ hearts. We found that SIRT1 protein levels were significantly repressed in cSIRT3^−/−^ hearts vs. SIRT3*^f/f^* hearts (Figure 1f). The results suggest that deficiency of SIRT3 blunted AMPK activation in aged and cSIRT3^−/−^ hearts during I/R stress because of SIRT1 reduction.

### 2.2 Cardiac SIRT1 and SIRT3 defects result in impaired cardiac function

**FIGURE 2:**
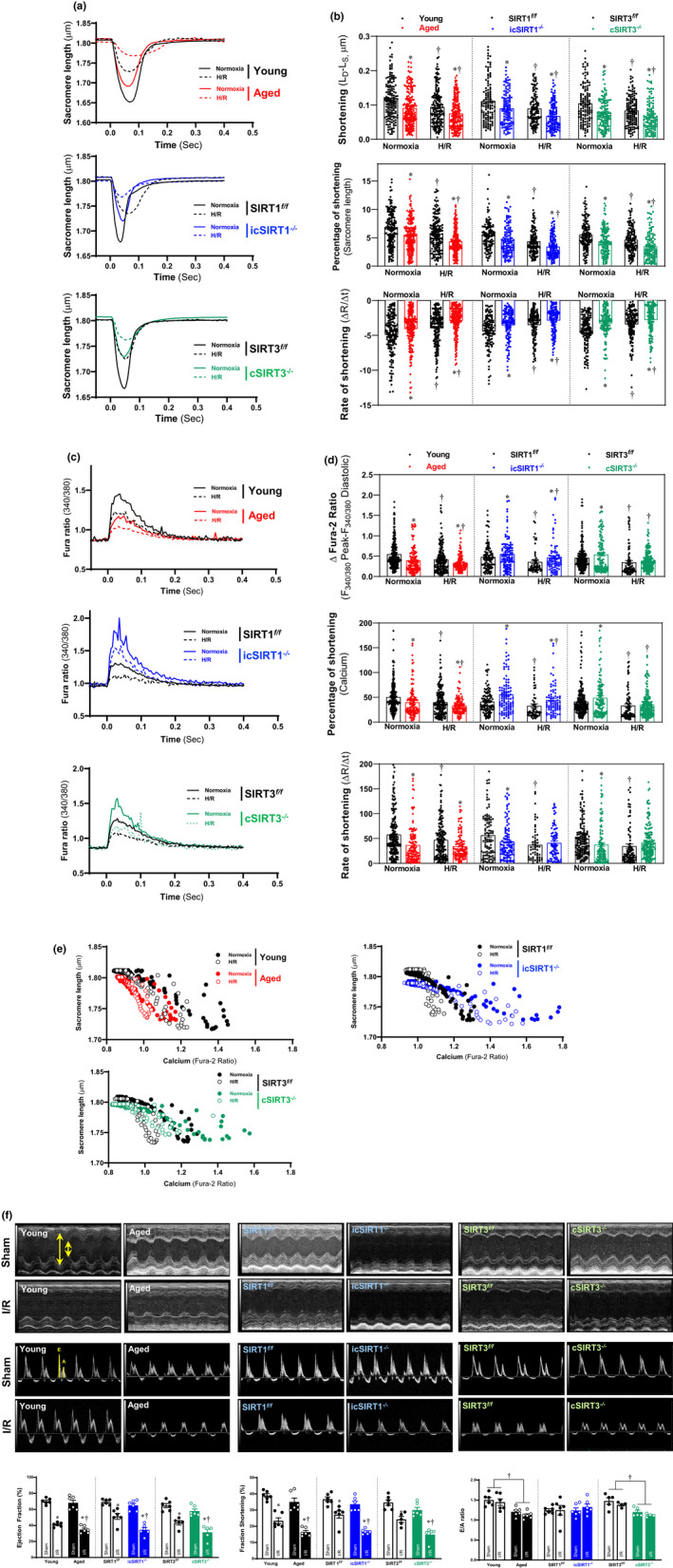
SIRT1 and SIRT3 are critical to maintaining the contractile properties of cardiomyocytes and heart in aging. (a) The representative cardiomyocyte contractility from young (4–6 months)/aged (24–26 months) WT, SIRT1*^f/f^* /icSIRT1^−/−^ (4–6 months), SIRT3*^f/f^*/cSIRT3^−/−^ (4–6 months) hearts. (b) The contractile properties of the isolated cardiomyocytes from young/aged WT, SIRT1*^f/f^*/icSIRT1^−/−^, and SIRT3*^f/f^*/cSIRT3^−/−^ hearts under normoxia and H/R stress conditions. Values are mean ± SEM. *N* ≥ 30 cardiomyocytes collected from 5 male mice for each group, *p < 0.05 vs. young, SIRT1*^f/f^*, SIRT3*^f/f^*, respectively: ^†^ *p* < 0.05 vs. normoxia, respectively. (c) The representative traces of transient calcium signal young/aged WT, SIRT1*^f/f^*/icSIRT1^−/−^, and SIRT3*^f/f^*/cSIRT3^−/−^ hearts. (d) The transient calcium signal response of the isolated cardiomyocytes from young/aged WT, SIRT1*^f/f^*/icSIRT1^−/−^, and SIRT3*^f/f^*/cSIRT3^−/−^hearts under normoxia and H/R stress conditions. Values are mean ± SEM. *N* ≥ 30 cardiomyocytes collected from 5 male mice for each group, *p < 0.05 vs. young, SIRT1*^f/f^*, SIRT3*^f/f^*, respectively: ^†^ *p* < 0.05 vs. normoxia, respectively. (e) The relationship loop between sarcomere length and calcium signal of the isolated cardiomyocytes from young/aged WT, SIRT1*^f/f^*/icSIRT1^−/−^, and SIRT3*^f/f^*/cSIRT3^−/−^ hearts under normoxia and H/R stress conditions. (f) Echocardiography showed that aged vs. young WT, icSIRT1^−/−^ vs. SIRT1*^f/f^* and cSIRT3^−/−^ vs. SIRT3*^f/f^* mice were more vulnerable to I/R stress as shown by ejection fraction (EF) and fractional shortening (FS). Upper: Reprehensive images of M‐mode echocardiography for systolic function and doppler of diastolic function. The yellow arrows and lines are the analysis method performed on Vevo Image software to obtain the parameters for systolic function and diastolic function analysis. Lower: Quantification of echocardiography measurements for EF, FS and E/A ratio. *N* ≥ 5, values are means ± SEM. *p < 0.05 vs. young, SIRT1*^f/f^*, SIRT3*^f/f^*, respectively; ^†^ *p* < 0.05 vs. sham, respectively

AMPK, as a central cellular sensor of energy metabolism, plays an important role in the regulation of myocardial signaling and contractile function in the aging heart (Dong et al., 2020; Turdi et al., 2010; Zaha et al., 2016). To further delineate whether the impaired AMPK activation caused by defected SIRT1 and SIRT3 precedes myocardial contractile dysfunction in the aged heart during I/R, we proposed to evaluate the contractile properties of isolated cardiomyocytes under normoxia or hypoxia/reoxygenation (H/R) conditions. During normoxia, compared with young cardiomyocytes, the contractile functions of the aged cardiomyocytes were observed by significantly reduced sarcomere shortening length, percentage of shortening and the rate of shortening (Figure 2a,b, upper panels). H/R stress impaired the contractile properties of young cardiomyocytes and the contractility became worse in the aged cardiomyocytes after H/R (Figure 2b). Cardiac deletion of SIRT1 leads to a limited cardiomyocyte contractility with the decline of three parameters as compared to the SIRT1*^f/f^* cardiomyocytes (Figure 2a,b, middle panels), which was even greater during H/R conditions (Figure 2a,b, middle panels). Moreover, the reduction in cardiomyocytes contractile properties was observed in cSIRT3^−/−^ cardiomyocytes as compared to SIRT3*^f/f^* cardiomyocytes under normoxia (Figure 2a,b, lower panels), the contractility became worse in both cSIRT3^−/−^ and SIRT3*^f/f^* under H/R stress (Figure 2a,b, lower panels). These data suggest that SIRT1 and SIRT3 are critical to maintain the contractile function of cardiomyocytes under physiological or pathological conditions.

Analysis of the transient calcium flux in cardiomyocytes by measuring the fura‐2 signal alterations showed that the aged cardiomyocytes reduced calcium flux in comparison with young cardiomyocytes with decreased three parameters (Figure 2c,d, upper panels). After H/R stress, the cardiomyocyte calcium flux declines in young cardiomyocytes, and this effect was exacerbated in aged cardiomyocytes during H/R (Figure 2c,d, upper panels). However, the calcium flux was elevated in icSIRT1^−/−^ cardiomyocytes during contraction with increased calcium shortening peak and the percentage of shortening as compared to the SIRT1*^f/f^* cardiomyocytes under normoxia (Figure 2c,d, middle panels). H/R stress also triggers the reduction of the two‐calcium flux‐related parameters in the icSIRT1^−/−^ cardiomyocytes but still in a high level of calcium flux vs. SIRT1*^f/f^* cardiomyocytes (Figure 2c,d, middle panels). However, the rate of shortening was decreased with the deletion of SIRT1 in cardiomyocytes under normoxia (Figure 2d). H/R stress causes the decline of the rate of shortening in the SIRT1*^f/f^* cardiomyocytes, which was blunted in icSIRT1^−/−^ cardiomyocytes (Figure 2d). Similarly, the calcium influx was elevated in cSIRT3^−/−^ cardiomyocytes during contraction with higher calcium shortening peak and the percentage of shortening as compared to the SIRT3*^f/f^* cardiomyocytes under normoxia (Figure 2c,d, lower panels). H/R stress triggers the reduction of the cardiomyocyte calcium influx in the cSIRT3^−/−^ cardiomyocytes accompanied by the decreased calcium shortening peak and the percentage of shortening (Figure 2c,d, lower panels). In addition, the rate of shortening was decreased with the deletion of SIRT3 in cardiomyocytes under normoxia and its reduction response caused by H/R stress was impaired in cSIRT3^−/−^ vs. to SIRT3*^f/f^* cardiomyocytes (Figure 2d, lower panel).

Moreover, the analysis of the contractile‐calcium flux relationship loop showed that H/R stress shifted the loop toward left and up indicating the reduction of contractile function and calcium flux in young cardiomyocytes, which is greater in aged cardiomyocytes (Figure 2e). The deletion of SIRT1 or SIRT3 in cardiomyocytes alters the loop shifted toward the right and up as compared to SIRT1*^f/f^* or SIRT3*^f/f^*, respectively indicating the reduction of contractile function and the increased calcium flux under normoxia, which tend to move left and up further after H/R stress in icSIRT1^−/−^ or cSIRT3^−/−^ cardiomyocytes (Figure 2e). These effects are consistent with the representative sarcomere length and calcium fura signal dynamic figures (Figure 2a,c), indicating that the age‐related deficiency of SIRT1 and SIRT3 in cardiomyocytes leads to the contractile dysfunctions and disturbed calcium flux during H/R.

In order to assess the effects of aged‐related SIRT1 and SIRT3 ablation on cardiac function, young (4–6 months)/aged (24–26 months) C57BL/6J mice, SIRT1*^f/f^*/icSIRT1^−/−^ (4–6 months) C57BL/6J mice, and SIRT3*^f/f^*/cSIRT3^−/−^ (4–6 months) C57BL/6J mice were subjected to sham operation or I/R surgery. The echocardiography measurements demonstrated that there are no significant differences in cardiac systolic functions among the groups under sham operations (Figure 2f, Table S1), but I/R stress (30 min of ischemia and 6 h of reperfusion) significantly reduced the systolic functions in all groups as shown by ejection fraction (EF) and fractional shortening (FS) (Figure 2f). Moreover, the aged vs. young, icSIRT1^−/−^ vs. SIRT1*^f/f^*, and cSIRT3^−/−^ vs. SIRT3*^f/f^* hearts are more sensitive to I/R stress as shown by the impaired EF and FS (Figure 2f). Interestingly, I/R stress did not affect cardiac diastolic function, while aged vs. young and cSIRT3^−/−^ vs. SIRT3*^f/f^* demonstrated a diastolic dysfunction as shown by reduction of E/A ratio (Figure 2f).

### 2.3 Cardiac deficiency of SIRT1 or SIRT3 impairs mitochondria OXPHOS complexes and respiration function during I/R stress

**FIGURE 3:**
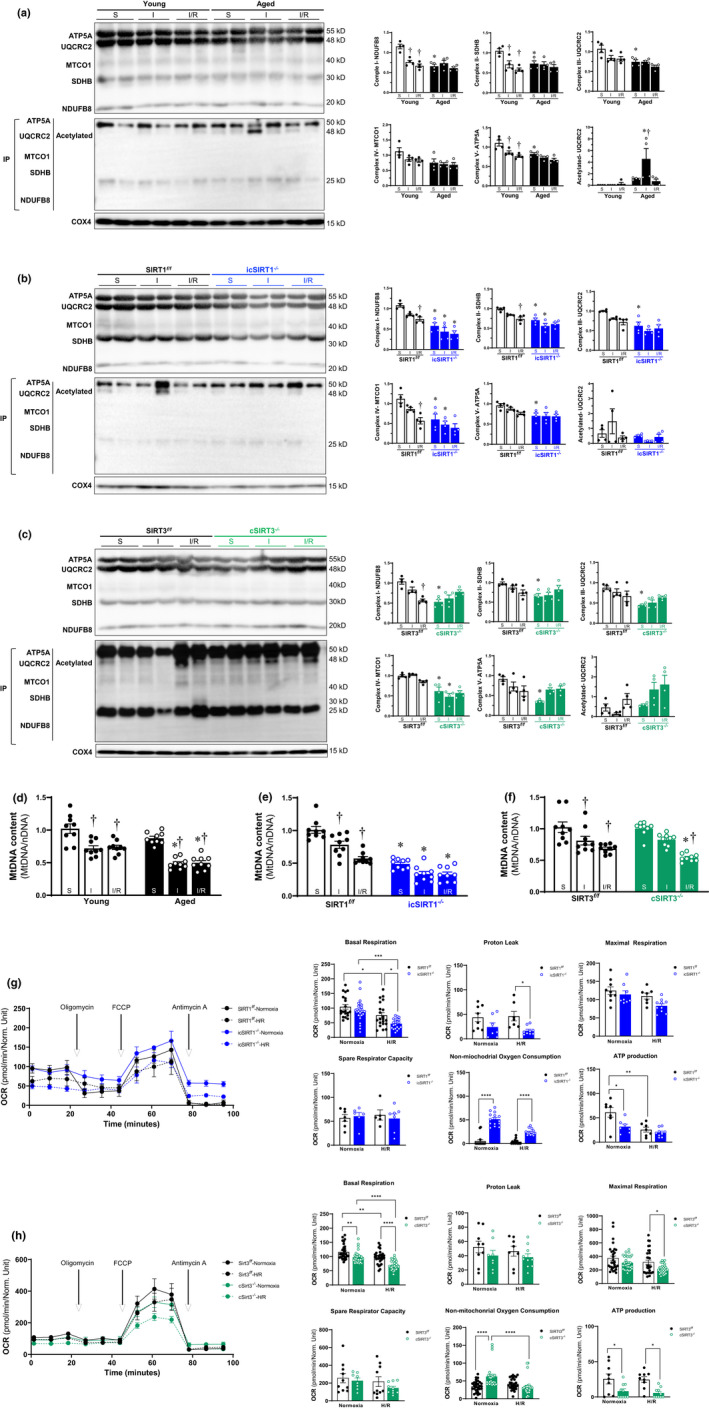
Deficiency of SIRT1 and SIRT3 in aging impaired the integrity of OXPHOS complexes under stress condition. (a‐c) Immunoprecipitation and immunoblotting analysis of OXPHOS complex I, II, III, IV, V subunits accumulation levels and acetylation in young (4–6 months)/aged (24–26 months) WT (a), SIRT1*^f/f^*/icSIRT1^−/−^ (4–6 months) (b), SIRT3*^f/f^*/cSIRT3^−/−^ (4–6 months) (c) hearts under physiological and I/R stress conditions. Values are mean ± SEM. *N* = 4, *p < 0.05 vs. young, SIRT1*^f/f^*, SIRT3*^f/f^*, respectively; ^†^ *p* < 0.05 vs. sham, respectively. (d‐f) Real‐time PCR measured the relative mitochondrial DNA (mtDNA) content (normalized to the nuclear gene) in the heart tissue of young/aged WT (d), SIRT1*^f/f^*/icSIRT1^−/−^ (e), SIRT3*^f/f^*/cSIRT3^−/−^ (f) hearts. Values are means  ± SEM, *n* = 9, ^†^ *p* < 0.05 vs. sham, respectively; *p < 0.05 vs. young, SIRT1*^f/f^*, SIRT3*^f/f^*, respectively; (g‐h) Mitochondrial respiration measurements of OCR in isolated cardiomyocytes of SIRT1*^f/f^*/icSIRT1^−/−^ (g), SIRT3*^f/f^*/cSIRT3^−/−^ (h) hearts were performed with a Seahorse metabolic analyzer. Oligomycin (1.5 μM), FCCP (1 μM), and antimycin (10 μM) were added sequentially to isolated cardiomyocytes under normoxia and H/R conditions. Quantitative analysis of mitochondrial function parameters (basal respiration, proton leak, maximal respiration, spare respiratory capacity, non‐mitochondrial oxygen consumption, and ATP production) is shown in the bar charts. Values are mean ± SEM, *n* ≥ 5, *p < 0.05 vs. SIRT1*^f/f^*, SIRT3*^f/f^*, respectively; ^†^ *p* < 0.05 vs. normoxia, respectively

Mitochondrial OXPHOS is the main fuel source of the heart, and the inability of energy generation has been considered the primary mechanism in linking mitochondrial dysfunction to cardiac contractile failure (Kuznetsov et al., 2019). To determine whether the downregulation of SIRT1 and SIRT3 with aging alter the cardiac mitochondrial function during I/R stress, we measured the integrity and acetylation of different subunits of mitochondrial OXPHOS complexes after LAD ligation or sham operations. The results demonstrated that the expression of majority subunits in Complex I subunit NDUFB8, Complex II subunit SDHB, and Complex V subunit ATP5A was impaired in the young heart during I/R stress (Figure 3a). However, the expression of majority subunits in Complex I, Complex II, Complex III, and Complex V was significantly downregulated in the aged WT vs. young WT hearts (Figure 3a), and their response to I/R stress in aged WT hearts was blunted as compared to young WT hearts (Figure 3a, upper panel). There was increased acetylation of complex III subunit UQCRC2 observed in aged (24–26 months) C57BL/6J hearts under ischemic condition compare with the young (4–6 months) C57BL/6J hearts (Figure 3a, lower panel). Interestingly, the protein levels of Complex I subunit NDUFB8, Complex II subunit SDHB, Complex III subunit UCQRC2, Complex IV MTCO1, and Complex V subunit ATP5A were significantly decreased with no significant change on the acetylation of these subunits in icSIRT1^−/−^ vs. SIRT1*^f/f^* hearts and lost the response to I/R stress in icSIRT1^−/−^ as compared to SIRT1*^f/f^* hearts (Figure 3b), suggesting that SIRT1 is required to maintain mitochondrial OXPHOS structural integrity instead of deacetylation of OXPHOS complex in the heart. A decline in OXPHOS Complex subunits was observed in cSIRT3^−/−^ vs. SIRT3*^f/f^* and the response effect of majority subunits in Complex I to Complex V to I/R stress were significantly impaired in cSIRT3^−/−^ vs. the SIRT3*^f/f^* hearts (Figure 3c). Higher acetylation of complex III subunit UQCRC2 was observed in cSIRT3^−/−^ hearts under I/R vs. sham but not reach statistical significance (Figure 3c). These results suggest that the impaired mitochondrial OXPHOS integrity and deacetylation are related to the reduction of SIRT3 in aging during I/R stress.

In addition, the accumulation of alterations in mtDNA affects the mitochondrial OXPHOS, because the mitochondrial genome is responsible for encoding the subunits of enzyme complexes involved in the biogenesis of the OXPHOS system (Chinopoulos, 2018). The quantitative analysis of mtDNA showed more damages that occurred in aged vs. young heart mitochondria during I/R (Figure 3d). The mtDNA content was decreased in icSIRT1^−/−^ vs. SIRT1*^f/f^* hearts (Figure 3e), and more mtDNA damage occurred in icSIRT1^−/−^ vs. SIRT1*^f/f^* hearts under I/R stress conditions (Figure 3e), indicating that SIRT1 is essential to maintain the mtDNA content under both physiological and pathological conditions. However, cSIRT3^−/−^ vs. SIRT3*^f/f^* hearts did not show any significant difference in the mtDNA content under sham condition but decreased under I/R condition (Figure 3f).

Considering the alterations of mitochondria OXPHOS complexes under I/R stress, the mitochondrial respiratory capacity of isolated cardiomyocytes from SIRT1*^f/f^* /icSIRT1^−/−^ hearts, and SIRT3*^f/f^*/cSIRT3^−/−^ hearts was measured under normoxia or H/R stress conditions. The results demonstrated that the oxygen consumption rate (OCR) of basal respiration and ATP production of SIRT1*^f/f^* cardiomyocytes were reduced during H/R vs. normoxia (Figure 3g). Significantly decreased basal respiration and ATP production were observed in icSIRT1^−/−^ vs. SIRT1*^f/f^* cardiomyocytes under normoxia (Figure 3g). Remarkably, H/R stress induces lower OCR of basal respiration and proton leak with increased non‐mitochondrial oxygen consumption in icSIRT1^−/−^ vs. SIRT1*^f/f^* cardiomyocytes (Figure 3g). Similarly, the results showed that the OCR of basal respiration of SIRT3*^f/f^* cardiomyocytes was significantly decreased during H/R vs. normoxia (Figure 3h). cSIRT3^−/−^ vs. SIRT3*^f/f^* showed an exacerbated mitochondrial dysfunction with impaired basal respiration, ATP production and non‐mitochondrial oxygen consumption during H/R (Figure 3h). These findings indicate that the age‐related deficiency of SIRT1 and SIRT3 in cardiomyocytes is associated with the mitochondria respiratory dysfunction during H/R stress.

### 2.4 Effects of SIRT1 and SIRT3 on mitochondria dynamics and redox homeostasis during I/R stress

**FIGURE 4:**
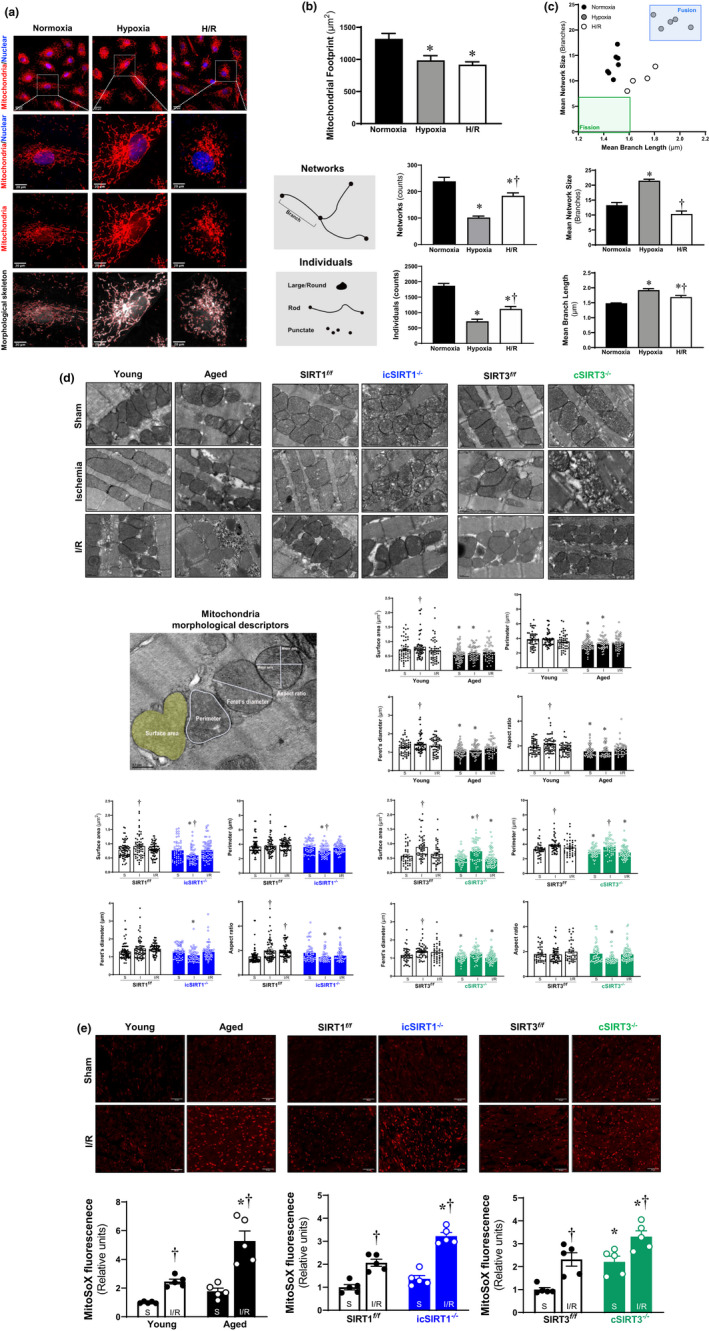
Alterations of mitochondria morphology induced by the age‐related decline of SIRT1 and SIRT3 under stress condition. (a) Representative images for H9c2 cells under normoxia and H/R stress conditions generated by applying the MiNA algorithm on the mitochondria stained with MitoTracker and DAPI. (b) Analysis of mitochondrial network morphology changes (mitochondria footprint, mitochondria individuals, and mitochondria networks) in H9c2 cells under normoxia and H/R stress condition using the MiNA macro for ImageJ. (c) Bar charts and relationship loop of mitochondria morphology parameters (mean network size and mean branch length) obtained from the analysis of 4–7 replicates for each group, with >10 cells counted for each replicate of H9c2 cells under normoxia and H/R stress conditions. *p < 0.05 vs. normoxia; ^†^ *p* < 0.05 vs. hypoxia condition. (d) Representative TEM images of in young (4–6 months)/aged (24–26 months) WT, SIRT1*^f/f^*/icSIRT1^−/−^ (4–6 months), and SIRT3*^f/f^*/cSIRT3^−/−^ (4–6 months) hearts under physiological and I/R stressed conditions. The images were analyzed using ImageJ to quantify the following morphological and shape descriptors: mitochondria surface area (µm^2^), perimeter (µm), Feret's diameter (µm), and aspect ratio. Values are means ± SEM. >50 mitochondria per group were measured. ^†^ *p* < 0.05 vs. sham, respectively; *p < 0.05 vs. young, SIRT1*^f/f^*, SIRT3*^f/f^*, respectively. (e) Representative microscopy images (200×) showing MitoSOX fluorescence in the myocardium in young/aged WT, SIRT1*^f/f^*/icSIRT1^−/−^, and SIRT3*^f/f^*/cSIRT3^−/−^ hearts under physiological and I/R stressed conditions (upper panel). Quantification of MitoSOX oxidation from images. Values are means ± SEM. *N* = 5 donors, ^†^ *p* < 0.05 vs. sham, respectively; *p < 0.05 vs young, SIRT1*^f/f^*, SIRT3*^f/f^*, respectively

Mitochondria, as highly dynamic structures organelle, have the capacity to adjust their morphology depending on energy demand and metabolic conditions in the majority of cells (Vasquez‐Trincado et al., 2016). The abnormality of mitochondrial dynamics contributes significantly to the pathological alterations of the heart including myocardial I/R (Dorn, 2015). To test the steady state of mitochondria, H9c2 cells were stained with MitoTracker and visualized by confocal microscopy. We observed the changes in mitochondrial morphology of H9c2 cells in response to H/R stress (Figure 4a). Using the MiNA script for mitochondrial network analysis in H9c2 cells in Fiji software (Valente et al., 2017), we can see a decrease of the mitochondrial footprint (means the total mitochondrial area) with a decreasing number of both mitochondria individuals and networks during H/R stress (Figure 4b). However, the latter observation found more frequent fusion related to the increase of larger tubular networks of mitochondria with elongated branches during H/R, perhaps relating the increased mitochondria mass caused by H/R stress (Figure 4c). These data confirmed that the alterations of mitochondrial dynamics contribute to cardiac mitochondria mass in response to hypoxic stress.

We also examined the ultrastructural changes of the mitochondria after I/R stress using transmission electron microscopy (TEM). The young (4–6 months) C57BL/6J mice heart exhibited healthy mitochondria with normal‐appearing cristae, and the mitochondria were elongated upon ischemic stress accompanied by increased mitochondria surface area, Feret's diameter and aspect ratio (Figure 4d). It suggests that ischemic stress triggers the processes of mitochondria fusion. However, the disrupted mitochondria with the decline of four shape descriptors in the aged (24–26 months) C57BL/6J vs. young hearts under the sham and greater impairment were observed in aged hearts during ischemia as compared to the young hearts (Figure 4d), indicating aging hearts lose the ability to regulate mitochondria dynamic, especially fusion, in response to I/R stress conditions (Figure 4d). Compared with SIRT1*^f/f^* hearts, significantly impaired cristae structure with brighter and diminished cristae membrane surface area was shown in icSIRT1^−/−^ hearts' mitochondria under sham condition (Figure 4d), and this case was worse during ischemic stress conditions (Figure 4d). The damaged mitochondria exhibited a significant decline of four shape descriptors in the icSIRT1^−/−^ vs. SIRT1*^f/f^* hearts under I/R stress (Figure 4d), suggesting that SIRT1 is required to mitochondria fusion in response to I/R stress. Similarly, the deletion of cardiomyocytes SIRT3 induced brighter and injured mitochondria cristae, while significant damage was observed upon ischemia accompanied by the decreased surface area, perimeter, and aspect ratio, indicating the elongation of mitochondria caused by ischemia was blunted in cSIRT3^−/−^ vs. SIRT3*^f/f^* hearts (Figure 4d). Taken together, these data indicate that the deficiency of SIRT1 and SIRT3 with aging contributes to the alterations of mitochondrial morphology during I/R stress.

I/R injury is associated with the release of extensive ROS as a result of abnormal mitochondrial homeostasis, especially in the aging population (Giorgi et al., 2018). SIRT1/SIRT3 is closely related to modulating the ROS production upon myocardial I/R stress (Parodi‐Rullan et al., 2017; Wang et al., 2018). In order to characterize the cardiac mitochondrial redox homeostasis, the intracellular ROS levels in the heart under physiological or I/R stress conditions were examined using MitoSox oxidation (Han et al., 2020). The results showed that myocardial I/R stress‐triggered ROS production in both young and aged hearts (Figure 4e, left panels), and more ROS levels in aged vs. young hearts under both sham and I/R stress conditions (Figure 4e, left panels). Moreover, icSIRT1^−/−^ vs. SIRT1*^f/f^* hearts displayed higher ROS levels under I/R stress condition (Figure 4e, middle panels). In addition, cSIRT3^−/−^ vs. SIRT3*^f/f^* hearts displayed higher ROS levels under shame condition, and I/R stress induced more ROS productions after I/R stress (Figure 4e, right panels). It suggests that age‐related deficiency in SIRT1 or SIRT3 alter cardiac intracellular redox homeostasis that causes more ROS production in the myocardium during I/R stress.

## 3 DISCUSSION

I/R stress is known to induce greater injury in the aged heart with no effective treatment strategy (Dong et al., 2020). The study demonstrated that both SIRT1 and SIRT3 were decreased with cardiac aging and age‐related deficiency of SIRT1 and SIRT3 plays critical role in maintaining cardiac function and cardiomyocyte contractile function in response to I/R stress.

SIRT1 is reported to be a novel therapeutic target for cardiovascular disease with effective deacetylase activity controlling multiple cellular processes (Han et al., 2017). We have reported that the age‐related deficiency of SIRT1 exacerbated cardiac systolic dysfunction with enlarged infarction size and metabolic disorder upon I/R stress (Tong et al., 2013; Wang et al., 2018). Our present study also confirmed the decline of SIRT1 with aging and the exacerbated cardiac dysfunction in aged and icSIRT1^−/−^ hearts during I/R validating the critical role of SIRT1 in protecting cardiac contractility from I/R injury in aging. However, the deletion of SIRT1 in cardiomyocyte induce elevated calcium flux during cardiomyocyte contraction, which could be a compensation mechanism or impaired calcium channel exchange system. Moreover, the deletion of SIRT1 in cardiomyocyte caused the hyperacetylation of LKB1 and impaired the phosphorylation of AMPK during ischemia (Wang et al., 2018). The compromised AMPK activation limited by SIRT1 reduction in aging during I/R stress suggests the significance of age‐related SIRT1 in regulating cardiac energy metabolism.

The deficiency of Sirtuin3 (SIRT3), the central control of mitochondrial protein deacetylation, aggravates the cardiac susceptibility to I/R stress with severe mitochondria abnormalities and exacerbates a higher level of myocardial I/R injury with aging (Parodi‐Rullan et al., 2017; Porter et al., 2014). Previous findings showed that SIRT3 played a protective role against mitochondrial damage via regulating the activation of energy metabolism regulator AMPK (Huh et al., 2016; Zhao et al., 2018). In this study, we found that SIRT3 declines with cardiac aging and SIRT3 is required to maintain the cardiomyocyte contractibility under physiological and pathological conditions. The significantly decreased contractile properties in cSIRT3^−/−^ mice were accompanied by the inactivation of AMPK signal during myocardial I/R injury, which is like aged wild type mice. These data suggesting that SIRT3 is critical to modulate AMPK to protect cardiac function in aging during myocardial I/R stress. In addition, SIRT3 deficiency might upregulate mitochondrial calcium uniporter (MCU) expression involved in AMPK regulation that accounts for exaggerated MCU‐mediated mitochondrial calcium uptake and resulting in the elevated calcium flux during cardiomyocyte contraction (Gao et al., 2020). Furthermore, the mechanism may involve in impairment of SIRT1‐mediated AMPK phosphorylation in response to cardiac I/R injury, because of the deletion of SIRT3 in cardiomyocytes induced the downregulation of SIRT1 expression.

Mitochondria are abundant in the heart and responsible for energy production for cardiomyocyte contractility (Lesnefsky & Hoppel, 2003). There are the potential reasons for the increased injury in the aged heart due to the compromised mitochondria respiration function and OXPHOS complexes (Lesnefsky & Hoppel, 2003). Our findings showed that mitochondrial respiratory defects in aging hearts during I/R stress that could be caused by the decline of SIRT1 and SIRT3 due to the significantly decreased of OXPHOS integrity and mtDNA content during myocardial I/R stress. Interestingly, the subunit UQCRC2 of Complex III was hyperacetylated in aged hearts and SIRT3 defected heart upon ischemic stress suggesting SIRT3 could contribute to this outcome during myocardial I/R stress as previous study (Novgorodov et al., 2016). Taken together, SIRT1 and SIRT3 play a critical role in limiting cellular damage by hypoxic or ischemic stress via modulating mitochondrial respiration.

Mitochondria are highly dynamic in structure, which responsible for generating the energy‐carrying molecule adenosine triphosphate (ATP) and modulating power‐related cellular processes in the heart (Maneechote et al., 2017; Vasquez‐Trincado et al., 2016). In current study, mitochondria network analysis showed decreased mitochondria area mean the impairment of mitochondria driven by hypoxic stress in H9c2 cells. Interestingly, those mitochondria have a larger network with longer branches suggesting the imbalanced mitochondria fission and fusion during H/R stress conditions. These findings indicating that the abnormality of mitochondrial morphology accounts for the cardiac cellular damage with H/R stress. The TEM data in the present study suggested that age‐related sensor SIRT1 and SIRT3 might contribute to the control of mitochondrial dynamic, especially mitochondria fusion, under I/R stress condition in the heart. Previous study found that aberrant mitochondrial dynamics is closely related to age‐related cardiac dysfunction with the deficiency of MFN1 and MFN2 (Chen et al., 2012; Nan et al., 2017; Papanicolaou et al., 2011, 2012), which was confirmed in this study that both MFN1 and MFN2 were downregulated in aged monkey hearts (Figure S1). It suggests that age‐related reduction of SIRT1 and SIRT3 may contribute to the alterations in mitochondria fusion related protein MFN1 and MFN2. Interestingly, the deacetylation of mitofusion‐related protein MFN1 and MFN2 by SIRT1 and OPA1 by SIRT3 are important for mitochondria survival (Lombard et al., 2007; Oanh et al., 2017; Parodi‐Rullan et al., 2018; Sooyeon et al., 2016). We believe that the deficiency of SIRT1 and SIRT3 in aging during I/R stress contributes to the higher acetylation of MFN1, MFN2 and OPA1, which mediated the alterations in mitochondria fusion. In addition, cells devoid of MFN1 and MFN2 displayed a fragmented mitochondrial morphology and increased the formation of mitochondria ROS (Papanicolaou et al., 2012). In our study, the augmented cardiac intracellular ROS level of aged, icSIRT1^−/−^, and cSIRT3^−/−^ mice demonstrated that SIRT1 and SIRT3 are critical to maintaining mitochondrial redox homeostasis with aging in response to myocardial I/R stress.

Our study does have some limitations. First, more detailed experiments are needed to interpret the mechanisms on how SIRT1 and SIRT3 specifically in cardiomyocytes prevent cardiac dysfunction via control the process of mitochondria dynamics. Secondly, given the remarkable cardiomyocyte‐specificity of these cellular events and their different physiological responses, further analysis of the functional effect of the age‐related deficiency of SIRT1 and SIRT3 in other cardiac cells is needed. Third, we performed the study in mice and monkeys, hampering our ability to explore and extend these findings to the patients. We advocate studying the role of SIRT1 and SIRT3 in ischemic heart disease populations to address future clinical usages and therapeutic options.

In summary, these data revealed age‐related SIRT1 and SIRT3 play critical roles in mitochondrial dynamics to maintain mitochondrial homeostasis in the heart under I/R stress, especially in the elderly population. The downregulation of cardiac SIRT1 and SIRT3 with aging impairs the integrity of OXPHOS components and mitochondrial respiration in response to I/R stress, which is detrimental to cardiac substrate metabolism accompanied by compromised AMPK activation. Thus, SIRT1 and SIRT3 are crucial to protect the age‐related cardiac contractile dysfunction during I/R stress.

## 4 EXPERIMENTAL PROCEDURES

### 4.1 Animals

Young (4–6 months) male C57BL/6J mice, SIRT1*^f/f^* (4–6 months) mice (stock number 008041), α‐MHC‐CreER^T2^ (4–6 months) (stock number 005657), SIRT3*^f/f^* (4–6 months) mice (stock number 031201), and α‐MHC‐Cre (4–6 months) mice (stock number 011038) were purchased from Jackson Laboratory. Aged (24–26 months) male C57BL/6J mice were supplied from Charles River Laboratories. Cardiomyocyte‐specific deletion of the SIRT1 gene mice was generated by breeding SIRT1*^f/f^* mice with transgenic mice that carried an autosomal integrated Cre gene driven by the cardiac‐specific alpha‐myosin heavy chain promoter (α‐MHC‐CreER^T2^). The inducible cardiac‐specific SIRT1 knockout (icSIRT1^−/−^) male mice were generated by Tamoxifen injection (0.08 mg/g, i.p. 5 days) of α‐MHC‐CreER^T2^‐SIRT1*^f/f^* (4–6 months) mice, and SIRT1*^f/f^* male mice (4–6 months) with Tamoxifen injection were used for control groups. Cardiomyocyte‐specific deletion of the SIRT3 (cSIRT3^−/−^) male mice (4–6 months) was generated by breeding SIRT3*^f/f^* mice with transgenic mice that carried an autosomal integrated Cre gene driven by the cardiac‐specific alpha‐myosin heavy chain promoter (α‐MHC‐Cre), and SIRT3*^f/f^* male mice (4–6 months) were used for control groups. The genotyping of mice was performed in the following way: genomic DNA was isolated with the Mouse Tail Quick Extraction kit (Bio Pioneer) from the tail. All animal protocols in this study were approved by the Institutional Animal Care and Use Committee of the University of South Florida as well as conform to the NIH Guide for the care and use of laboratory animals.

### 4.2 In vivo regional ischemia/reperfusion

Mice were anesthetized, intubated, and ventilated as we previously described (Han et al., 2020). Mice were anesthetized with 2%–3% Isoflurane and placed on a heating pad to maintain body temperature at 37°C. After a left lateral thoracotomy, the left anterior descending coronary artery (LAD) was occulated for 30 mins with an 8–0 nylon suture and polyethylene tubing to prevent arterial injury and subsequently reperfused for 6 h. ECGs were utilized to confirm the ischemic hallmark of the ST‐segment elevation during coronary occlusion (AD Instruments). At the end of reperfusion, the hearts were excised, and left ventricles were separated before freeze clamping in liquid nitrogen.

### 4.3 Immunoprecipitation and Immunoblotting

Immunoblots and immunoprecipitation were performed as previously describe (Han et al., 2020; Wang et al., 2018). For immunoprecipitation analysis, 500 μg lysates were mixed with antibodies at 4°C 1 h followed by the addition of 40 μl of Protein A/G PLUS‐Agarose (Santa Cruz) at 4°C overnight. Immune complexes were washed three times with lysis buffer and then boiling. Equivalent amounts of protein samples (20 μg per lane) were subjected to SDS/PAGE. Total rodent OXPHOS cocktail antibody (ab110413) and rabbit polyclonal antibodies against SIRT1 (ab12193) from Abcam (MitoSciences‐Abcam, Eugene, OR). SIRT3 (#5490), AMP‐activated protein kinase α (AMPK α) (#2532), phosphor‐AMPKα (Thr^172^) (#2535), and GAPDH (#2118), from Cell signaling and used according to protocols provided by the manufacturer.

### 4.4 Cardiomyocytes isolation

Heparin IV (Fresenius Kabi) for anticoagulation was given by intraperitoneal injection with 1000 units/kg 10 min before the experiment (Li et al., 2019; Sun et al., 2020). Mice underwent anesthesia with 2%–3% isoflurane and 100% O_2_. The hearts of mice were excised, then cannulated by the aorta and connected to the cardiomyocyte perfusion apparatus (Radnoti). The heart was perfused at 37°C with a Ca^2+^ free based buffer (pH 7.2) containing: 135 mM NaCl, 4 mM KCl, 1 mM MgCl_2_, 10 mM HEPES, 0.33 mM NaH_2_PO_4_, 10 mM glucose, 10 mM 2, 3‐butanedione monoxime, and 5 mM taurine that was bubbled with O_2_. The heart was digested with 0.03 mg/ml Liberase (Sigma, # 5401020001) dissolved in perfusion buffer. After digested completely, the heart was removed, torn with tweezers and blown gently, then filtered to get the isolated cardiomyocytes.

### 4.5 Measurement of contractility of the cardiomyocytes

The contractile properties of cardiomyocytes were assessed by an IonOptix Multi Cell High Throughput system (IonOptix Corporation). Cardiomyocytes were placed in a chamber and stimulated with a 14‐voltage at a frequency of 1 Hz. IonWizard software was used to record the changes in sarcomere length and duration of shortening and relengthening. The following parameters were used to evaluate cardiomyocytes contractile property: maximum change of sarcomere length during contraction (Shortening (L_D_‐L_S_)); and the percentage of shortening; the maximum velocity of shortening (ΔL/Δt).

### 4.6 Intracellular Ca^2+^ transient measurement

Intracellular Ca^2+^ was measured using a dual‐excitation, single emission photomultiplier system (IonOptix) (Li et al., 2019; Lu et al., 2020). Cardiomyocytes were treated with Fura 2‐AM (2 μM) at 37°C for 20 min and then exposed to light emitted by a 75 W halogen lamp through either a 340‐ or 380‐nm filter while being stimulated to contract at 14 voltages with a frequency of 1 Hz. Fluorescence emissions were detected. The following parameters were recorded: maximum changes of calcium signal during contraction (ΔFura Ratio); the maximum velocity of shortening (ΔR/Δt); and the percentage of shortening.

### 4.7 Relative quantification of mitochondria DNA copy number

Total DNA was isolated from the hearts via QIAamp^®^ DNA Mini Kit (QIAGEN). The mtDNA content relative to nuclear DNA was assessed by real‐time qPCR (QuantStudio™ 3). Relative mitochondria DNA (mtDNA) content was determined using the ΔCT method. Primers of the mtDNA 16S rRNA were forward, 5′‐CTAGAAACCCCGAAACCAAA‐3′; reverse, 5′‐CCAGCTATCACCAAGCTCGT‐3′. Primers of the nuclear gene Ndufv1 were forward, 5′‐CTTCCCCACTGGCCTCAAG‐3′; reverse, 5′‐CCAAAACCCAGTGATCCAGC‐3′.

### 4.8 Mitochondrial respiration measurements

The Seahorse XF24 was used to measure the oxygen consumption rate (OCR) of isolated cardiomyocytes. Isolated cardiomyocytes were differentiated in customized Seahorse 24‐well plates and treated with hypoxia and reoxygenation as described above. After treatment, the medium was replaced with DEME Medium (Seahorse Bioscience), supplemented with 1 mM pyruvate, 2 mM glutamine, and 10 mM D‐glucose. OCR was measured using the Seahorse Bioscience XF24 Extracellular Flux Analyzer (Seahorse Bioscience). Measurements were taken as the cells were incubated sequentially under four conditions: (1) basal levels were measured with no additives; (2) oligomycin (1.5 μM) was added to reversibly inhibit ATP synthase and OXPHOS, showing glycolysis alone; (3) FCCP (1 μM), a mitochondrial uncoupler, was added to induce maximal respiration; and (4) Antimycin A (10 μM), a Complex I inhibitor and mitochondrial poison, was added to end the reaction. The Seahorse software was used to plot the results. OCR was normalized to cell numbers per well.

### 4.9 Cell culture and reagents

Rat cardiac myoblast H9c2 cells (ATCC CRL‐1446™) were cultured in high glucose (4.5 g/L) DMEM (Corning Cellgro) supplemented with 10% (v/v) fetal bovine serum (FBS) (Gibco. Life Technologies) and antibiotics (100 U/mL penicillin and 100 μg/ml streptomycin) (ATCC). The cells were maintained in a humidified incubator with 95% air and 5% CO_2_ at 37°C.

### 4.10 Hypoxia and reoxygenation (H/R) treatment

H9c2 cells or isolated cardiomyocytes were placed in a nitrogen chamber containing 95% N_2_/5% CO_2_ for 12 h or 20 min to induce hypoxia, respectively (Li et al., 2019; Lu et al., 2020). The cardiomyocytes were then removed from the nitrogen chamber and allowed to reoxygenate for 6 h and 20 min under normal atmospheric oxygen levels, respectively. Normoxic cardiomyocytes were placed at room temperature under normal atmospheric oxygen levels.

### 4.11 Mitotracker staining and quantitative analysis of mitochondrial morphology

For staining mitochondria, H9c2 cells were treated with hypoxia and reoxygenation as described above and then incubated with MitoTracker Deep Red FM (M22426, Molecular Probes) at a final concentration of 200nM for 15 min at 37°C. Finally, slides were washed with PBS three times and stained with 4′,6'‐diamidino‐2‐phenylindole (DAPI; Sigma‐Aldrich, D9542) for 5 min at room temperature. Cells were mounted onto slides and observed on Olympus FV1200 (MPE) confocal microscope. Analysis of mitochondrial network morphology in individual cells was done using the MiNA tool for the opensource software Fiji (ImageJ) (Valente et al., 2017). 2D microscopy images were minimally processed using the “unsharp mask” included in the Fiji package to enhance sharpness and then binarized to allow running the remaining features of the MiNA script, namely skeletonizing followed by measurement of networks (branched structures), individuals, mitochondrial coverage area (mitochondrial footprint).

### 4.12 Transmission electron microscope and mitochondria dynamics analysis

Heart tissues were rapidly immersed in McDowell's Trump Fixative (Electron Microscopy Science) at 4°C for 48 h and then trimmed to 1 mm^3^ in size. After the trim, the block was thin sectioned (90–100 nm thick) and then applied to copper grids and air dried. The grid was loaded in a JEOL1400 transmission electron microscopy (Jeol). At least 5 randomly selected electron micrographs of longitudinally arranged cardiomyocytes from each sample were examined. The images were analyzed using ImageJ to quantify the following morphological and shape descriptors: mitochondria surface area (µm^2^), perimeter (µm), Feret's diameter (longest distance (µm) between any two points within a given mitochondrion, aspect ratio (major axis)/(minor axis), the measure of the “length to width ratio”).

### 4.13 ROS measurements

MitoSOX Red (Invitrogen) was used to measure mitochondrial reactive oxygen species (ROS) production. Freshly frozen sections of LV were washed by PBS and then incubated within PBS containing 1 μM MitoSOX™ Red mitochondrial superoxide indicator (Invitrogen) for 15 min at 37°C, protected from light. Images were detected by fluorescence microscopes (excitation at 510, emission at 580 nm).

### 4.14 Statistics analysis

The analysis results of cardiac function, superoxide accumulation, and immunoblotting were expressed as means ± standard error of the means (SEM). Two‐tailed Student's *t* test, one‐way ANOVA with Tukey's test, and Kruskal–Wallis test were used to perform the comparison of the statistics among a set of samples with Prism 8.0 (GraphPad Software). *p* < 0.05 was considered a significant difference.

## CONFLICT OF INTEREST

The authors declare that they have no conflict of interest.

## AUTHOR CONTRIBUTIONS

J. Zhang, D. Ren, and J. Li designed, conducted the study, and wrote the manuscript; J. Zhang, D. Ren, J. Fedorova, Z. He, C. Logan, L. Bates, K. Davitt, M. Li, M. Wang, E.G. Lakatta, and J. Li performed data collection, analysis, and interpreted data.

## Supporting information

Supplementary Material

## Data Availability

The data that support the findings of this study are available on request from the corresponding author.
