# Supplementary material for: Alterations in mitochondrial dynamics with age‐related Sirtuin1/Sirtuin3 deficiency impair cardiomyocyte contractility
Source: Aging Cell. 2021 Jul 3;20(7):e13419. doi: 10.1111/acel.13419 (PMC8282250; doi:10.1111/acel.13419)
Supplement: Supplementary file 1 — Supplementary Material [file ACEL-20-e13419-s001.pdf]

Alterations in mitochondrial dynamics with age-related sirtuin1/sirtuin3 deficiency impair cardiomyocyte contractility

Jingwen Zhang | Zhibin He | Julia Fedorova |Cole Logan | Lauryn Bates | Kayla Davitt | Van Le | Jiayuan Murphy | Melissa Li | Mingyi Wang | Edward G. Lakatta | Di Ren | Ji Li

1 | Supplementary Methods

1.1 | Nonhuman primate studies

The snap-frozen Left ventricular tissue for western blotting was harvested from young (n =4, a range of age: from 8.87 to 14.12 yrs; average = 10.84 yrs) and aged (n=5, a range of age: from 18.81 to 26.48 yrs; average=22.4 yrs) male rhesus monkeys (Macaca mulatta) from NIA. All these monkeys were on a standard diet (CD, Harlan, Cat. No. TD 02498) (http://grantome.com/grant/NIH/ZIA-AG000238-09) (M. Wang, Lakatta, E.G., 2019)

1.2 | Immunoblotting

Immunoblotting was performed as previously described. SIRT1 (H300) from Santa cruz Biotechnology, Inc was used for monkey samples, and rabbit polyclonal antibodiey against SIRT1 (ab12193) from Abcam (MitoSciences-Abcam, Eugene, OR) was used for H9c2 cell samples. SIRT3 (#5490), OPA1 (#80471), Drp1 (#8570), and GAPDH (#2118) from Cell signaling (Danvers, MA). COXIV (COX4) (NB110-39115), mitofusion1 (MFN1) (NBP1-51841) and mitofusion2 (MFN2) (NBP2-17298) from Novus Biologicals (Littleton, CO) were purchased and used according to protocols provided by the manufacturer.

1.3 | Transient transfection with small interfering RNA (siRNA)

To knockdown the endogenous SIRT1 and SIRT3, H9c2 cells were transiently transfected with 100 nM siRNAs targeting SIRT1 (Ambion s174220), SIRT3 (sc-61556), or with the non-silencing control siRNA (scram) (Ambion) using Lipofectamine™ RNAiMAX (Invitrogen) in culture medium without antibiotics according to the manufacturer's recommendations.

1.4 | Mitochondrial membrane potential (MMP) measurement

The mitochondrial membrane potential (MMP) of H9c2 cells was assessed with a JC-1 mitochondrial membrane potential assay kit (Abcam, ab113850) according to the manufacturer's instructions. The cells were incubated with 5 μM JC-1 dye for 20 minutes at 37°C and then washed twice with 1X dilution buffer. The samples were assessed on a fluorescent plate reader (BioTek). For red fluorescence, the fluorescence intensity was measured at Ex/Em: 525/590 nm. The green fluorescence intensity was measured at Ex/Em: 490/530 nm.

1.5 | Mitochondrial respiration measurements

The Seahorse XF96 was used to measure the oxygen consumption rate (OCR). Transfected H9c2 cells (6,000/well) were differentiated in customized Seahorse 96-well plates and treated with hypoxia and reoxygenation as described above. After treatment, the medium was replaced with XF Assay Medium (Seahorse Bioscience), supplemented with 1 mM pyruvate, 2 mM glutamine, and 10 mM D-glucose. After placing the cell culture microplate into a 37°C non-CO2 incubator for 1 hour, OCR was measured using the Seahorse Bioscience XF96 Extracellular Flux Analyzer (Seahorse Bioscience). Measurements were taken as the cells were incubated sequentially under four conditions: (1) basal levels were measured with no additives; (2) oligomycin (1 μM) was added to reversibly inhibit ATP synthase and OXPHOS, showing glycolysis alone; (3) FCCP (2 μM), a mitochondrial uncoupler, was added to induce maximal respiration; and (4) Rotenone/antimycin A (0.5 μM), a Complex I inhibitor and mitochondrial poison, was added to end the reaction. The Seahorse software was used to plot the results. OCR was normalized to cell protein concentration per well.

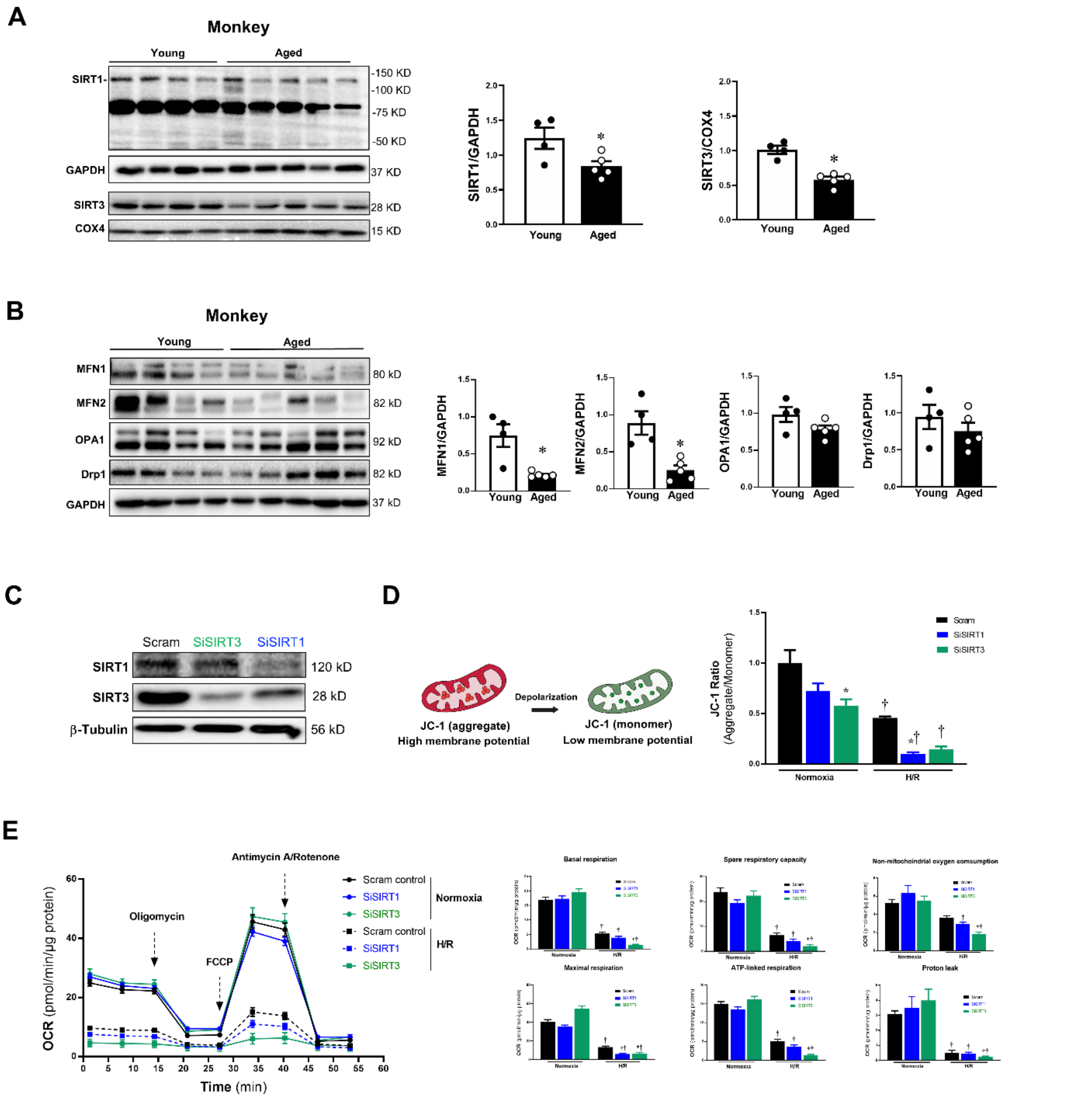

Suppl Figure 1. Deficiency of SIRT1 and SIRT3 cause alterations in mitochondria homeostasis with aging in monkey hearts and under H/R stress in H9c2 cells. (A) Immunoblotting showed the protein expression levels of SIRT1 and SIRT3 were decreased with aging in the monkey hearts under physiological condition. Values are mean ± SEM. N=4-5, *p<0.05 *vs.* young. (B) Immunoblotting showed the expression level of mitochondria dynamics-related protein MFN1, MFN2 was decreased with aging in the monkey whole hearts under the physiological condition with unchanged OPA1 and Drp1 expression. Values are mean ± SEM. N=4-5, *p<0.05 *vs.* young. (C) The representative protein expression level of SIRT1 and SIRT3 in H9c2 cells treated with SIRT1 siRNA or SIRT3 siRNA. (D) The mitochondrial membrane potential was evaluated by JC-1 staining. Values are mean ± SEM. Three independent experiment for each group, with N>=3 replicates, *p<0.05 *vs.* scram; †p<0.05 *vs.* normoxia, respectively. (E) Mitochondrial respiration measurements of OCR were performed with a Seahorse metabolic analyzer. Oligomycin (1 μM), FCCP (2 μM), and rotenone (0.5 μM) combined with antimycin (0.5 μM) were added sequentially to H9c2 cells treated with SIRT1 siRNA or SIRT3 siRNA under normoxia and H/R conditions. Quantitative analysis of mitochondrial function parameters (basal respiration, maximal respiration, spare respiratory capacity, ATP-linked respiration, non-mitochondrial oxygen consumption and proton leak) are shown in the bar charts. Values are mean ± SEM. N>=3 biological replicates, *p<0.05 *vs.* scram; †p<0.05 *vs.* normoxia, respectively.

Suppl Table 1. Echocardiographic measurements of mouse hearts functions under either sham or ischemia (30 min)/reperfusion (6 h) (I/R) conditions.

Group	**Young** (4-6 months) (C57BL/6J)		**Aged** (24-26 months) (C57BL/6J)		**SIRT1$^{f/f}$** (4-6 months) (C57BL/6J)		**icSIRT1$^{-/-}$** (4-6 months) (C57BL/6J)		**SIRT3$^{f/f}$** (4-6 months) (C57BL/6J)		**cSIRT3$^{-/-}$** (4-6 months) (C57BL/6J)	
	**Sham**	**I/R**	**Sham**	**I/R**	**Sham**	**I/R**	**Sham**	**I/R**	**Sham**	**I/R**	**Sham**	**I/R**
**HR**	413.45±36.02	426.45±34.06	399.67±12.05	412.79±67.19	427.43±43.78	396.08± 19.03	392.36±27.68	428.37±20.31	403.00±34.24	437.11±79.82	388.45±45.17	431.25±78.72
**CO**	14.58±2.60	11.22±4.07*	13.99±2.38	10.94±2.91*	14.57±3.33	13.58±3.74	12.78±3.36†	11.41±1.68†	15.35±2.45	11.64±5.89*	14.42±3.43	9.95±5.93*†
**LV Mass**	121.06±25.55	133.12±29.23	164.39±30.03	145.04±26.96	137.27±30.49	160.7±65.73	184.53±56.89	168.52±43.09	126.95±15.61	126.39±9.63	126.23±9.09	137.53±12.59
**SV**	35.79±8.70	26.48±9.92	34.91±5.20	27.50±9.45	36.00±7.56	34.9±7.06	32.97±6.89	26.68±4.04	38.13±5.50	27.16±12.79	37.82±0.94	23.26±13.21
**LVAW;s**	1.31±0.19	1.16±0.30	1.48±0.18	1.11±0.19	1.35±0.20	1.39±0.37	1.42±0.15	1.30±0.36	1.18±0.16	1.18±0.14	1.26±0.16	1.32±0.18
**LVAW;d**	0.82±0.12	0.90±0.18	1.09±0.18	0.85±0.18	0.94±0.14	0.98±0.31	1.10±0.15	1.10±0.28	0.81±0.10	0.92±0.05	0.86±0.12	1.07±0.16
**LVPW;s**	1.41±0.24	1.12±0.24	1.49±0.25	1.19±0.47	1.52±0.16	1.51±0.47	1.51±0.30	1.35±0.36	1.16±0.16	1.19±0.12	1.21±0.11	1.45±0.44
**LVPW;d**	0.97±0.19	0.91±0.20	1.14±0.24	0.99±0.39	1.09±0.15	1.13±0.41	1.19±0.20	1.07±0.29	0.85±0.16	0.94±0.18	0.87±0.17	1.13±0.28
**Volume;s**	23.39±10.40	41.12±13.28	24.73±7.76	47.44±15.93	17.28±6.17	26.45±11.7	30.85±15.14	37.37±14.20	35.71±12.12	31.56±5.92	30.74±7.92	23.34± 8.81
**Volume;d**	59.18±16.56	67.6±22.78	59.64±9.36	74.94±23.56	53.28±9.16	61.35±10.65	63.82±18.08	64.05±15.55	73.84±15.73	58.72±13.83	68.56±16.41	46.6±17.29
**Diameter;s**	2.49±0.50	3.17±0.45	2.58±0.35	3.36±0.51	2.23±0.32	2.63±0.46	2.74±0.73	3.04±0.50	2.99±0.42	2.87±0.22	2.83±0.31	2.50±0.44
**Diameter;d**	3.70±0.46	3.89±0.61	3.73±0.24	4.07±0.58	3.56±0.26	3.77±0.28	3.81±0.50	3.83±0.41	4.07±0.37	3.69±0.37	3.94±0.40	3.33±0.53

Note: HR, Heart rate; **CO,** Cardiac output; **SV,** Stroke volume; **IVAW;s**, Left ventricular end-systolic anterior wall thickness (systolic); **IVAW;d,** Left ventricular end-diastolic anterior wall thickness (diastolic); **IVCT,** Isovolumic contraction time; **LVPW;s**, Left ventricular posterior wall (systolic); **LVPW;d,** Left ventricular posterior wall (diastolic); **Volume;s**, Left ventricular end-systolic volume (systolic); **Volum;s**, Left ventricular end-diastolic volume (diastolic); **Diameter;s,** left ventricular internal dimension at end systole; **Diameter;s,** left ventricular internal dimension at end diastole. N=6, Values are expressed as mean ± SD. *p<0.05 vs. sham, †p<0.05 vs. young, SIRT1$^{f/f}$, SIRT3$^{f/f}$, respectively.
